# Smart Dual‐Exsolved Self‐Assembled Anode Enables Efficient and Robust Methane‐Fueled Solid Oxide Fuel Cells

**DOI:** 10.1002/advs.202306845

**Published:** 2023-11-20

**Authors:** Feng Hu, Kongfa Chen, Yihan Ling, Yonglong Huang, Sunce Zhao, Sijiao Wang, Liangqi Gui, Beibei He, Ling Zhao

**Affiliations:** ^1^ Faculty of Materials Science and Chemistry China University of Geosciences Wuhan 430074 China; ^2^ College of Materials Science and Engineering Fuzhou University Fuzhou Fujian 350108 China; ^3^ School of Materials Science and Physics China University of Mining and Technology Xuzhou 221116 China; ^4^ School of Materials Science and Engineering Jingdezhen Ceramic University Jingdezhen 333403 China; ^5^ Zhejiang Institute China University of Geosciences (Wuhan) Hangzhou 311305 China; ^6^ Shenzhen Research Institute China University of Geosciences Shenzhen 518000 China

**Keywords:** anodes, dual‐exsolution, hydrocarbons, self‐assembly, solid oxide fuel cells

## Abstract

Perovskite oxides have emerged as alternative anode materials for hydrocarbon‐fueled solid oxide fuel cells (SOFCs). Nevertheless, the sluggish kinetics for hydrocarbon conversion hinder their commercial applications. Herein, a novel dual‐exsolved self‐assembled anode for CH_4_‐fueled SOFCs is developed. The designed Ru@Ru‐Sr_2_Fe_1.5_Mo_0.5_O_6‐δ_(SFM)/Ru‐Gd_0.1_Ce_0.9_O_2‐δ_(GDC) anode exhibits a unique hierarchical structure of nano‐heterointerfaces exsolved on submicron skeletons. As a result, the Ru@Ru‐SFM/Ru‐GDC anode‐based single cell achieves high peak power densities of 1.03 and 0.63 W cm^−2^ at 800 °C under humidified H_2_ and CH_4_, surpassing most reported perovskite‐based anodes. Moreover, this anode demonstrates negligible degradation over 200 h in humidified CH_4_, indicating high resistance to carbon deposition. Density functional theory calculations reveal that the created metal‐oxide heterointerfaces of Ru@Ru‐SFM and Ru@Ru‐GDC have higher intrinsic activities for CH_4_ conversion compared to pristine SFM. These findings highlight a viable design of the dual‐exsolved self‐assembled anode for efficient and robust hydrocarbon‐fueled SOFCs.

## Graphical abstract

To achieve efficient and robust CH_4_ fueled solid oxide fuel cells, a hierarchical Ru@Ru‐Sr_2_Fe_1.5_Mo_0.5_O_6‐δ_ (SFM)/Ru‐Gd_0.1_Ce_0.9_O_2‐δ_ (GDC) anode is developed by an innovative integration of self‐assembly and dual exsolution. The single cell using this anode delivers a high peak power density of 0.63 W cm^−2^ at 800 °C and a remarkable stability for 200 h using humidified CH_4_ as fuel.

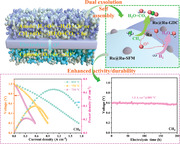

## 1 Introduction

To counteract worsening climate change and mounting energy shortfalls, solid oxide fuel cells (SOFCs) have emerged as a promising power generation technology because of their high efficiency and eco‐friendliness.^[^ ^1^ ^]^ Unlike low‐temperature fuel cells, SOFCs operating at high temperatures offer a distinct feature of fuel flexibility, allowing direct conversion of the chemical energy in available hydrocarbons, ammonia, and complex fuels into electricity.^[^ ^2^ ^]^ However, owing to high activity for catalyzing the cleavage of C−H bonds, traditional Ni‐based ceramic anodes of SOFCs readily suffer from carbon coking when directly using hydrocarbons as fuels, causing the decreased active sites and the corresponding performance degradation.^[^ ^3^ ^]^ Moreover, the volume change during redox cycles and the Ni coarsening during the long‐term operation of Ni‐based ceramic anodes also restrict the energy efficiency and operating life of SOFCs.^[^ ^4^ ^]^

In response, enormous efforts have been devoted to developing alternative anode materials to Ni‐based cermets for hydrocarbon‐fueled SOFCs. Particularly, perovskite oxides (general formula of ABO_3_)^[^ ^5^ ^]^ with mixed ionic‐electronic conduction, such as doped‐SrTiO_3‐δ_ (ST),^[^ ^6^ ^]^ La_0.75_Sr_0.25_Cr_0.5_Mn_0.5_O_3‐δ_ (LSCM),^[^ ^7^ ^]^ PrBaMn_2_O_5+δ_ (PBM)^[^ ^8^ ^]^ and Sr_2_Fe_1.5_Mo_0.5_O_6‐δ_ (SFM),^[^ ^9^ ^]^ and their derivatives, show great potentials as anodes for hydrocarbon fueled SOFCs because of their high carbon deposition‐resistance and excellent structural stability. Nevertheless, the sluggish kinetics of perovskite oxides toward hydrocarbon conversion, which are typically lower than the common Ni‐based ceramic anodes, restricts their large‐scale commercialization in SOFCs.

Actually, developing a single‐phase material that meets all the requirements of the anode for hydrocarbon‐fueled SOFCs is challenging. Instead, the rational design of composite electrodes, where each component serves its specific functionality, is likely a more promising approach. For example, combining perovskite oxide with an oxygen ionic conductor (e.g., doped CeO_2_) not only facilitated the oxygen ionic transfer but also stabilized the interfacial contact of anode/electrolyte, eventually resulting in a remarkable decrease in polarization resistance.^[^ ^10^ ^]^ Constructing a metal‐oxide heterointerface represents another viable approach to promote the intrinsic activity of electrocatalysts. Particularly, in situ exsolution has emerged as an advanced strategy to precisely manipulate the metal‐oxide heterointerface,^[^ ^11^ ^]^ compared to the conventional infiltration method involving multiple and unmanageable deposition and heat treatment processes.^[^ ^12^ ^]^ Of paramount significance, the exsolved heterointerface showcases distinctive advantages, including metal‐oxide heterointerface for synergistic electrocatalysis, the strong metal‐oxide interaction for curtailing carbon deposition and metal nanoparticles agglomeration, and reversible structure during redox cycling.^[^ ^13^ ^]^ For instance, the single cell with a CoFe exsolved Sr_2_Fe_1.5‐x_Co_x_Mo_0.5_O_6‐δ_ anode demonstrated a peak power density of 0.27 W cm^−2^ at 800 °C for 130 h using CH_4_ as the fuel.^[^ ^14^ ^]^ A RuFe exsolved SrTi_0.3_Fe_0.7_Ru_0.07_O_3‐δ_ anode showed a high coking resistance in steam/ethanol mixtures.^[^ ^15^ ^]^ The crucial descriptors involved in regulating the morphology and performance of exsolved heterointerface have been revealed, including exsolved metal component determined by exsolution energy, defect tailoring of perovskite host, and phase transformation of perovskite host et al.^[^ ^16^ ^]^ Therefore, introducing oxygen ionic conductor and constructing a metal‐oxide heterointerface are effective solutions to boost the performance of perovskite anodes. However, developing a heterogeneous anode with well‐tailored components and morphology to simultaneously achieve high intrinsic activity and fast oxygen ionic conduction is a significant challenge.

Herein, we highlight a smart dual‐exsolved self‐assembled anode enabling efficient and robust CH4‐fueled SOFCs. The initial heterogeneous anode, comprising Ru incorporated Sr_2_Fe_1.5_Mo_0.5_O_6‐δ_ (SFM) and Ru incorporated Gd_0.1_Ce_0.9_O_2‐δ_ (GDC) phases, is self‐assembled by a one‐pot method. It has been reported that Ru element demonstrates reversible dissolution and exsolution capabilities in both SFM perovskite^[^ ^17^ ^]^ and CeO_2_ fluorite^[^ ^18^ ^]^ lattices. As expected, Ru metal is in situ exsolved on both Ru‐SFM and Ru‐GDC surfaces induced by a reducing atmosphere during the actual operation of SOFCs, creating Ru@Ru‐SFM/Ru‐GDC dual‐exsolved self‐assembled anode. Unlike conventional mechanical mixing composite anodes, the self‐assembled anode promises an enlarged three‐phase boundary benefitting electrocatalysis.^[^ ^19^ ^]^ Furthermore, leveraging the cooperation of oxygen ionic conductor and the creation of exsolved heterointerfaces, the single cell using the Ru@Ru‐SFM/Ru‐GDC anode delivers high peak power densities of 1.03 and 0.63 W cm^−2^ at 800 °C under humidified H_2_ and CH_4_, far exceeding the referenced SFM/GDC anode. Using humidified CH_4_ as fuel, this anode demonstrates remarkable durability for 200 h without apparent carbon deposition. As revealed by DFT calculations, the heterointerfaces of Ru@Ru‐SFM and Ru@Ru‐GDC exhibit smaller energy barriers compared to SFM, denoting higher intrinsic activities for CH_4_ conversion.

## 2 Results and Discussion

### 2.1 Morphology and Structure

**Figure 1:**
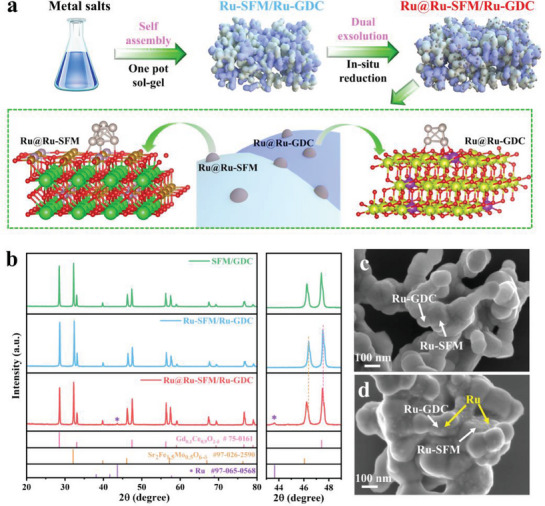
a) Schematic illustration of structure evolution of dual‐exsolved self‐assembled Ru@Ru‐SFM/Ru‐GDC electrocatalyst, b) powder XRD patterns of SFM/GDC, Ru‐SFM/Ru‐GDC and Ru@Ru‐SFM/Ru‐GDC electrocatalysts, SEM images of c) Ru‐SFM/Ru‐GDC and d) Ru@Ru‐SFM/Ru‐GDC electrocatalysts.

**Figure** **1**a illustrates the structural change for the formation of the dual‐exsolved self‐assembled Ru@Ru‐SFM/Ru‐GDC electrocatalyst. Using a facile one‐pot method, mixed metal salts are self‐assembled into Ru‐incorporated SFM and Ru‐incorporated GDC phases. Notably, when the anode exposed to a reducing atmosphere in actual operating, metallic Ru is segregated onto both Ru‐SFM and Ru‐GDC surfaces simultaneously, receiving the Ru@Ru‐SFM/Ru‐GDC anode. Figure 1b provides the X‐ray diffraction (XRD) patterns of SFM/GDC, Ru‐SFM/Ru‐GDC and Ru@Ru‐SFM/Ru‐GDC electrocatalysts. Similar to SFM/GDC electrocatalyst, the Ru‐incorporated Ru‐SFM/Ru‐GDC electrocatalyst exhibits a composition comprising a cubic perovskite phase and a cubic fluorite phase.^[^ ^20^ ^]^ Characteristic peaks corresponding to individual RuO_2_ are absent, suggesting the potential Ru doping into the SFM and GDC lattices. After reduction, an additional peak (≈43.7°) emerges in the Ru@Ru‐SFM/Ru‐GDC electrocatalyst, well indexed to the phase of Ru (PDF #97‐065‐0568). Concurrently, shifts in the peaks representative of the perovskite and fluorite phases toward lower angles are observed, implying that the lattice expansion is likely induced via the presence of low‐valence metal cations and the generation of oxygen vacancies after reduction. As shown in hydrogen temperature programmed reduction (H_2_‐TPR, Figure S1, Supporting Information), an obvious hydrogen consumption peak ≈282 °C likely corresponds to the exsolution of metallic Ru.

To gain insights into the morphology of the Ru‐SFM/Ru‐GDC and Ru@Ru‐SFM/Ru‐GDC electrocatalysts, scanning electron microscopy (SEM) and high‐resolution transmission electron microscopy (HRTEM) analysis were performed. The SEM image reveals a smooth surface of submicron Ru‐SFM/Ru‐GDC particles. (Figure 1c). After being exposed to a reducing atmosphere, large amounts of nano‐sized particles are decorated on the surface of the Ru‐SFM/Ru‐GDC backbone (Figure 1d). Analysis of the HRTEM image for Ru‐SFM/Ru‐GDC (Figure S2, Supporting Information) indicates lattice spacing of 0.226 and 0.163 nm, which are well indexed to the (220) plane of Ru‐SFM and (311) plane of Ru‐GDC, respectively. Energy dispersive spectrometer (EDS) mapping further demonstrates that the anode self‐assembles into Ru‐SFM and Ru‐GDC phases, and the Ru element is uniformly distributed in the Ru‐SFM and Ru‐GDC backbones.

**Figure 2:**
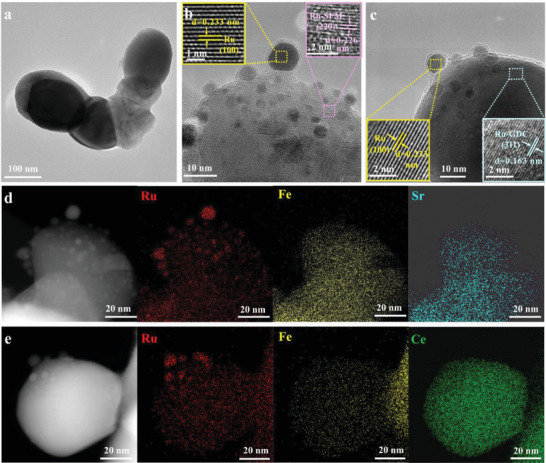
a) HRTEM image of total Ru@Ru‐SFM/Ru‐GDC electrocatalyst, b) selected Ru@Ru‐SFM particle, c) selected Ru@Ru‐GDC particle, EDS mapping of d) selected Ru@Ru‐SFM particle, e) selected Ru@Ru‐GDC particle.

In contrast, a collection of spherical nanoparticles is deeply anchored onto the surfaces of Ru‐SFM/Ru‐GDC backbones after reduction (**Figure** **2**a). Of note, this strongly anchored configuration induced by in situ exsolution promises a potent metal‐oxide interaction that offers the potential to inhibit carbon deposition and the agglomeration of exsolved nanoparticles. Beyond Ru‐SFM (220) and Ru‐GDC (311) planes in the backbone, the lattice spacing of surface exsolved nanoparticles is 0.233 nm, fairly agreeing with the (100) plane of metallic Ru (Figure 2b,c). In addition, the average particle sizes of exsolved Ru nanoparticles on Ru‐SFM and Ru‐GDC surfaces are ≈2.73 and ≈3.62 nm (Figure S3, Supporting Information), respectively. EDS elemental distribution mapping analysis further validates the presence of Ru@Ru‐SFM (Figure 2d) and Ru@Ru‐GDC (Figure 2e) heterointerfaces. These results affirm that the fabrication of Ru@Ru‐SFM/Ru‐GDC electrocatalyst has been successfully achieved through the integrated methodology of self‐assembly and dual‐exsolution.

**Figure 3:**
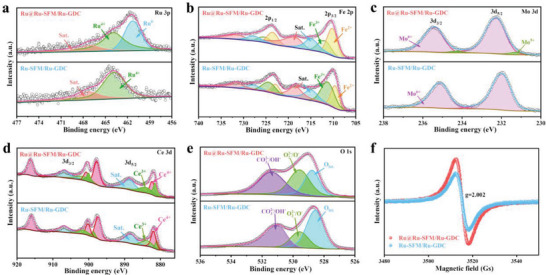
XPS spectra of a) Ru 3p, b) Fe 2p, c) Mo 3d, d) Ce 3d, e) O 1s, and f) EPR spectra of Ru‐SFM/Ru‐GDC and Ru@Ru‐SFM/Ru‐GDC electrocatalysts.

To assess the influence of exsolution on elemental chemical states, X‐ray photoelectron spectroscopy (XPS) measurements were conducted on Ru‐SFM/Ru‐GDC and Ru@Ru‐SFM/Ru‐GDC electrocatalysts. **Figure** **3**a shows that the peak leak centered at 463.8 eV is well assigned to the Ru^4+^ state in the Ru‐SFM/Ru‐GDC electrocatalyst.^[^ ^17^ ^]^ It is noteworthy that a new peak located at 461.3 eV is recorded in Ru@Ru‐SFM/Ru‐GDC electrocatalyst, confirming the exsolution of metallic Ru after reduction. Contrasting outcomes are observed for other metal ions such as Fe (Figure 3b), Mo (Figure 3c), and Ce (Figure 3d), where their reduced average valence states are established post reduction (Table S1, Supporting Information), accompanied by the absence of their metallic states. For instance, the Fe^3+^:Fe^2+^ valence ratio in Ru‐SFM/Ru‐GDC reduces from 57.5%:42.5% to 39.0%:61.0% in Ru@Ru‐SFM/Ru‐GDC. These findings bolster the contention that the exsolved particles predominantly constitute metallic Ru rather than alloy.

The reduction in metal ion valence state always corresponds with the generation of oxygen vacancies to maintain electrical neutrality. The O 1s XPS spectra of Ru‐SFM/Ru‐GDC and Ru@Ru‐SFM/Ru‐GDC electrocatalysts are categorized into the oxygen species of carbonate/hydroxyl oxygen (CO_3_^2−^/OH^−^) centered at 531.2 eV, adsorbed oxygen (O_2_^2−^/O^−^) centered at 529.5 eV, lattice oxygen (O_lat._) centered at 528.5 eV (Figure 3e).^[^ ^21^ ^]^ The elevated adsorbed oxygen: lattice oxygen ratio following reduction indicates a higher concentration of oxygen vacancies in the Ru@Ru‐SFM/Ru‐GDC electrocatalyst compared to Ru‐SFM/Ru‐GDC (Table S1, Supporting Information). Furthermore, electron paramagnetic resonance (EPR) analysis reveals a heightened peak intensity for Ru@Ru‐SFM/Ru‐GDC (Figure 3f), also indicating a greater abundance of oxygen vacancies than in Ru‐SFM/Ru‐GDC.^[^ ^22^ ^]^ The created oxygen vacancies facilitate rapid oxygen mobility and contribute to the oxidative conversion of hydrocarbons.^[^ ^23^ ^]^ Remarkably, the structure change is reversible during redox cycles. As confirmed by XRD, SEM, and XPS (Figures S4–S6 and Table S2, Supporting Information), the exsolved Ru nanoparticels are redissolved into Ru‐SFM/Ru‐GDC lattice after reoxidation, and Ru nanoparticels can be exsolved again after rereduction (Figures S7–S9 and Table S2, Supporting Information). Such good reversibility of exsolution/dissolution suggests the structural flexibility of dual‐exsolved self‐assembled electrocatalyst.

### 2.2 Enhanced Electrochemical Performance

Electrochemical impedance spectra (EIS) of anodes were examined using symmetric cells (Figure S10, Supporting Information) at 800 °C in 5% H_2_‐Ar atmosphere, to comprehend the impact of Ru dual‐exsolution on electrochemical processes. As shown in Figure S11, Supporting Information, the Ru@Ru‐SFM/Ru‐GDC anode exhibits smaller polarization resistances (*R*_p_) and lower activation energy compared to the SFM/GDC anode. This suggests an accelerated reaction kinetics for H_2_ oxidation after dual‐exsolution. Furthermore, distribution of relaxation time (DRT) analysis exhibits that the impedance spectra consist of distinct high‐frequency polarization (HF), medium‐frequency polarization (MF), and low‐frequency polarization (LF) arcs, corresponding respectively to charge transfer, ion surface exchange and transmission, and gas absorption/desorption processes.^[^ ^24^ ^]^ Both LF‐R_p_ and MF‐R_p_ significantly decreases, implying that the ion surface exchange and transmission, and gas absorption/desorption are accelerated by dual‐exsolution of metallic Ru.

**Figure 4:**
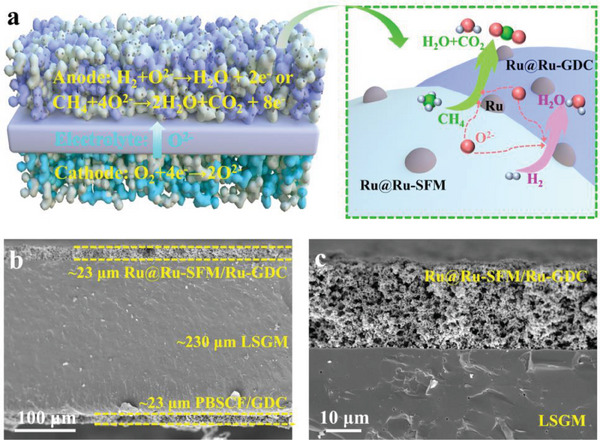
a) Schematic diagram illustrating H_2_ or CH_4_ fueled SOFCs, SEM cross‐sectional views of b) electrolyte supported single cell and c) Ru@Ru‐SFM/Ru‐GDC anode.

To evaluate the practical viability of the Ru@Ru‐SFM/Ru‐GDC electrocatalyst as a potential anode for SOFCs, single SOFCs with an asymmetric architecture of porous Ru@Ru‐SFM/Ru‐GDC (anode) || dense LSGM (electrolyte) || porous PBSCF/GDC (cathode) were prepared (**Figure** **4**a). For comparison, SOFCs employing a SFM/GDC anode were also investigated. The compositional purities of the synthetic LSGM electrolyte and PBSCF/GDC composite cathode are confirmed by XRD analysis (Figure S12, Supporting Information). As depicted in Figure 4b, the Ru@Ru‐SFM/Ru‐GDC anode, ≈23 µm in thickness, and the PBSCF/GDC cathode, ≈23 µm in thickness, are both screen‐printed on opposing sides of a ≈230 µm thick LSGM electrolyte. Remarkably, using the smart self‐assembly approach results in the fabrication of a submicron‐structured Ru‐SFM/Ru‐GDC skeleton (Figure 4c). The following in situ dual‐exsolution of nanoscale metallic Ru onto such submicron skeleton yields a hierarchical anode structure characterized by abundant heterointerfaces conducive to electrocatalytic activity. According to BET analyses (Table S3, Supporting Information), the dual‐exsolved Ru‐SFM/Ru‐GDC anode exhibits a higher surface area of 8.80 m^2^ g^−1^ than that of unexsolved SFM/GDC anode (6.38 m^2^ g^−1^). Additionally, the robust contact in the interface of electrolyte and anode ensures high structural stability and rapid ion transport.

**Figure 5:**
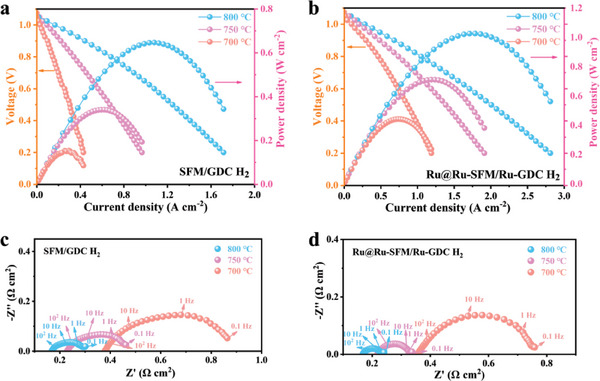
I−V and I−P curves of the H_2_ fueled single cells using a) SFM/GDC anode and b) Ru@Ru‐SFM/Ru‐GDC anode, EIS curves of the H_2_ fueled single cells using c) SFM/GDC anode and d) Ru@Ru‐SFM/Ru‐GDC anode.

Using humidified H_2_ (≈3% H_2_O) as the fuel, the peak power densities (PPD) of 0.65, 0.34, and 0.16 W cm^−2^ are achieved on the SFM/GDC single cell at operating temperatures of 800, 750, and 700 °C (**Figure** **5**a). In contrast, the Ru@Ru‐SFM/Ru‐GDC single cell exhibits superior performance, with PPD values of 1.03, 0.71, and 0.44 W cm^−2^ at 800, 750, and 700 °C (Figure 5b), respectively. This enhanced performance of the Ru@Ru‐SFM/Ru‐GDC single cell can be primarily attributed to its lower polarization resistance (*R*_p_) compared to the SFM/GDC single cell (0.073 vs. 0.134 Ω cm^2^ at 800 °C), as demonstrated in Figure 5c,d. In view of almost the same electrolyte and cathode materials, the reduced R_p_ indicates that the anodic reaction kinetics associated with H_2_ oxidation is significantly enhanced by dual‐exsolution, in line with the results of symmetrical cells. In addition, compared to single cells using other high performing perovskite anodes, the PPD of the single cell featuring the Ru@Ru‐SFM/Ru‐GDC anode surpasses most of the reported ones (Table S4, Supporting Information).

**Figure 6:**
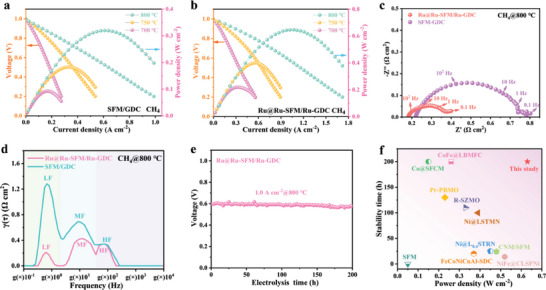
*I−V* and *I−P* curves of the CH_4_ fueled single cells using a) SFM/GDC anode and b) Ru@Ru‐SFM/Ru‐GDC anode, c) EIS curves and d) DRT analysis, e) cell potential as function of elapsed time for the Ru@Ru‐SFM/Ru‐GDC single cell, f) comparison of the peak power densities of CH_4_ fueled single cells (Sr_2_Fe_1.5_Mo_0.5_O_6–δ_ (SFM),^[^ ^9a^ ^]^ Sr_2_ZnMoO (R‐SZMO),^[^ ^25^ ^]^ Co@Sr_2_Fe_1.3_Co_0.2_Mo_0.5_O_6‐δ_ (Co@SFCM),^[^ ^26^ ^]^ CoNiMo/Sr_2_FeMoO_6–δ_ (CNM/SFM),^[^ ^27^ ^]^ CoFe@La_0.5_Ba_0.5_Mn_0.8_Fe_0.1_Co_0.1_O_3‐δ_ (CoFe@LBMFC),^[^ ^28^ ^]^ Pr_6_O_11_‐PrBaMn_2_O_5+δ_ (Pr‐PBMO),^[^ ^29^ ^]^ Ni@La_0.4_Sr_0.4_Ti_0.85_Ru_0.07_Ni_0.08_O_3−δ_ (Ni@L0.4STRN),^[^ ^30^ ^]^ Ni@(La_0.2_Sr_0.8_)_0.925_Ti_0.55_Mn_0.35_Ni_0.1_O_3‐δ_ (Ni@LSTMN),^[^ ^31^ ^]^ NiFe@La_0.6_Ce_0.1_Sr_0.3_Fe_0.9_Ni_0.1_O_3‐δ_ (NiFe@CLSFNi),^[^ ^32^ ^]^ FeCoNiCuAl‐Sm_0.2_Ce_0.8_O_2_ (FeCoNiCuAl‐SDC)^[^ ^33^ ^]^).

When transitioning to the direct utilization of humidified CH_4_ (≈3% H_2_O), the PPD of the SFM/GDC single cell is 0.32 W cm^−2^ at 800 °C (**Figure** **6**a), while the Ru@Ru‐SFM/Ru‐GDC single cell achieves a higher PPD of 0.63 W cm^−2^ at the same temperature (Figure 6b). The R_p_ of the Ru@Ru‐SFM/Ru‐GDC single cell (0.22 Ω cm^2^ at 800 °C) is remarkably lower than that of the SFM/GDC single cell (0.58 Ω cm^2^ at 800 °C) (Figure 6c), denoting that the Ru@Ru‐SFM/Ru‐GDC anode offers a much higher electrocatalytic activity toward CH_4_ conversion. According to DRT analysis, three characteristic peaks in the HF, MF, and LF ranges are identified (Figure 6d). Among them, the LF‐R_p_ is dramatically reduced, indicative of the significantly enhanced CH_4_ absorption/dissociation kinetics after Ru dual‐exsolution.^[^ ^33^ ^]^

### 2.3 Carbon Deposition Tolerance

**Figure 7:**
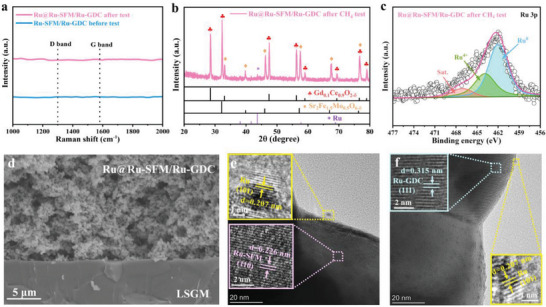
a) Raman spectrum, b) XRD pattern, c) XPS Ru 3p spectrum, d) SEM image, TEM images of e) selected Ru@Ru‐SFM particle and f) selected Ru@Ru‐GDC particle for the Ru@Ru‐SFM/Ru‐GDC anode after long‐term durability testing.

The galvanostatic test of the Ru@Ru‐SFM/Ru‐GDC anode based single cell was conducted at 800 °C, applying a constant current density of 1.0 A cm^−2^. As depicted in Figure 6e, the cell voltage remains consistently stable at ≈0.59 V for 200 h. Of note, the PPD and durability of the CH_4_ fueled single cell using the Ru@Ru‐SFM/Ru‐GDC anode favorably rival most of previously reported perovskite anodes, as illustrated in Figure 6f and Table S5 (Supporting Information). No Raman carbon peaks, including the D‐band at 1357 cm^−1^ and G‐band at 1585 cm^−1^, are detected on the Ru@Ru‐SFM/Ru‐GDC anode after the extensive long‐term stability test (**Figure** **7**a), demonstrating the exceptional resistance of the Ru@Ru‐SFM/Ru‐GDC anode to carbon deposition during CH_4_ exposure. Moreover, the original phase structure of the Ru@Ru‐SFM/Ru‐GDC anode is almost not changed after long‐term CH_4_ operation, as shown in Figure 7b. XPS analyses in Figure 7c and Figure S13 (Supporting Information), confirm the consistent existence of metallic Ru within the Ru@Ru‐SFM/Ru‐GDC anode.

In addition, the morphology of the Ru@Ru‐SFM/Ru‐GDC anode after long‐term durability test was investigated by SEM and TEM. This self‐assembled anode retains its highly porous submicron structure without the presence of discernible carbon deposits (Figure 7d). It is worth noting that the exsolved nanoparticles firmly anchor onto the Ru‐SFM/Ru‐GDC substrate without agglomeration, as shown in Figure 7e,f. These findings indicate that the nano‐heterointerfaces in the Ru@Ru‐SFM/Ru‐GDC anode maintain excellent structural stability throughout CH_4_ operation, likely owing to the strong metal‐support interaction created by the exsolution approach.^[^ ^31^ ^]^

### 2.4 Mechanism of CH_4_ Conversion

**Figure 8:**
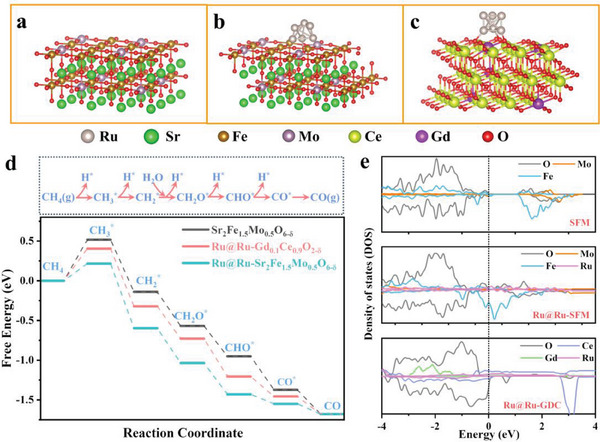
Theoretical modes of pristine a) SFM, b) Ru@Ru‐SFM, and c) Ru@Ru‐GDC, d) free‐energy profiles of CH_4_ conversion, e) density of states for SFM, Ru@Ru‐SFM, and Ru@Ru‐GDC.

Experimental characterizations have substantiated the occurrence of dual exsolution, resulting in the formation of Ru@Ru‐SFM and Ru@Ru‐GDC heterointerfaces, which significantly improve the activity of CH_4_ conversion. Consequently, we conducted density functional theory (DFT) calculations to elucidate the underlying mechanism governing CH_4_ conversion over dual‐exsolved heterointerfaces. Theoretical modes of pristine SFM (001), Ru cluster@Ru‐SFM (001), and Ru cluster@Ru‐GDC (111) are built, as depicted in **Figure** **8**a–c, respectively. A widely accepted pathway and corresponding free energy profiles of CH_4_ conversion are illustrated in Figure 8d.^[^ ^34^ ^]^ The relevant reaction intermediates adsorbed on SFM, Ru@Ru‐SFM, and Ru@Ru‐GDC are respectively depicted in Figures S14–S16 (Supporting Information). These calculations conclusively establish that the CH_4_ activation as the formation of CH_3_* is the rate‐determining step. Existing literatures had also corroborated that the initial dehydrogenation process was mainly the rate determining step for hydrocarbon conversion.^[^ ^23^, ^25^ ^]^ Afterward, the CH_4_ conversion process shows a downhill energy trend. It is evident that the calculated energy barriers of Ru@Ru‐SFM (0.22 eV), and Ru@Ru‐GDC (0.40 eV) heterointerfaces are lower than that of pristine SFM (0.52 eV), endowing them with higher intrinsic activities toward CH_4_ conversion.

Figure 8e provides the calculated partial density of states (DOS) of SFM, Ru@Ru‐SFM, and Ru@Ru‐GDC. One can see that the specific orbital contributions of Ru@Ru‐SFM, and Ru@Ru‐GDC heterointerfaces around the Fermi level are richer than that of pristine SFM. Compared with the pristine SFM, the Ru@Ru‐SFM, and Ru@Ru‐GDC heterointerfaces substantially facilitate the CH_4_ adsorption/activation by effectively weakening the C−H bond,^[^ ^23^ ^]^ promoting the activities toward CH_4_ conversion. Moreover, it has been revealed that the synergistic effect of Lewis acid (perovskite oxide) and Lewis base (exsolved metal) contributes to CH_4_ dehydrogenation process.^[^ ^35^ ^]^ Herein, the Lewis acid‐Lewis base pairs of exsolved Ru@Ru‐SFM perovskite oxide and exsolved Ru@Ru‐GDC fluorite oxide^[^ ^36^ ^]^ follow the same mechanism.

Beyond high intrinsic activities of heterointerfaces, the Ru@Ru‐SFM/GDC anode, prepared by an integrated approach combining self‐assembly and dual‐exsolution, exhibits a nano@submicron hierarchical structure, ensuring a high density of heterointerfaces. Furthermore, the introduction of GDC can enhance the oxygen ionic conductivity of the anode, significantly expanding the electrochemical active area from the anode/electrolyte near‐interface to the anode bulk.^[^ ^37^ ^]^ This combination of high intrinsic activity, high density of active sites, and high oxygen ionic conduction establishes Ru@Ru‐SFM/Ru‐GDC as a prominent anode material with outstanding electrochemical performance for CH_4_ fueled SOFCs.

## 3 Conclusion

In summary, we have developed a novel Ru@Ru‐SFM/Ru‐GDC anode of SOFCs by a dual‐exsolution and self‐assembly approach, which delivers remarkable activity for CH_4_ conversion and high tolerance to carbon deposition. The Ru@Ru‐SFM/Ru‐GDC anode based single cell delivers a high peak power density of 0.63 W cm^−2^ at 800 °C and a remarkable durability for 200 h with humidified CH_4_ as fuel, favorably rivalling most reported perovskite‐based anodes for CH_4_ fueled SOFCs. Experimental findings and DFT calculations reveal that the high electrochemical performance is primarily attributed to the unique hierarchical architecture with high oxygen ionic conduction, rich heterointerfaces and high intrinsic activity with low energy barrier for CH_4_ conversion. The innovative integration of self‐assembly and dual exsolution in the Ru@Ru‐SFM/Ru‐GDC anode offers a promising solution for high‐performance hydrocarbon fueled SOFCs, which might also guide the design of heterointerfaces for various electrocatalysis.

## 4 Experimental Section

### Powder Preparation

The raw materials were analytically pure and were purchased by Sinopharm Chemical Reagent Co., Ltd. Ru@Ru‐Sr_2_Fe_1.5_Mo_0.5_O_6‐δ_(SFM)/Ru‐Gd_0.1_Ce_0.9_O_2‐δ_(GDC) anode was prepared by an elegant one‐pot self‐assembly approach. The molar doping levels of pretailored Ru in SFM and GDC were 10% and 5%, respectively. The mass ratio of Ru‐SFM: Ru‐GDC was ≈6:4. Citric acid monohydrate and glycolic acid as complexing agents were first introduced into deionized water, followed by introducing stoichiometric quantities of Sr(NO_3_)_2_, Fe(NO_3_)_3_•9H_2_O, (NH_4_)_6_Mo_7_O_24_•4H_2_O, Gd(NO_3_)_3_•6H_2_O, Ce(NO_3_)_3_•6H_2_O, and Ru(NO_3_)•xH_2_O. The obtained homogeneous precursor solution was stirred continuously at ≈80 °C for 3 h to generate a viscous gel. The gel was then heat‐treated at 300 °C for 1 h in an oven to obtain a spongy powder. Such precursor was calcined in air at 1100 °C for 3 h to acquire the self‐assembled Ru‐SFM/Ru‐GDC composite anode. The dual‐exsolved self‐assembled Ru@Ru‐SFM/Ru‐GDC composite anode was in situ created via the emergence of Ru metal nanoparticles on the surface of Ru‐SFM/Ru‐GDC substrate during H_2_ or CH_4_ fuel cell operation at high temperatures. Additionally, La_0.8_Sr_0.2_Ga_0.8_Mg_0.2_O_3‐δ_ (LSGM) electrolyte, PrBa_0.5_Sr_0.5_Co_1.5_Fe_0.5_O_5+δ_ (PBSCF)/GDC cathode, and compared SFM/GDC anode were prepared using the similar one‐pot method.

### Cell Fabrication and Measurement

Electrolyte‐supporting single cell with a sandwich structure of Ru‐SFM/Ru‐GDC anode || LSGM electrolyte || PBSCF/GDC cathode was fabricated. The LSGM disk was prepared by dry‐pressing and sintering at 1400 °C for 5 h in air, with the thickness of ≈230 µm for the single cell. The anode and cathode inks were separately obtained by dispersing terpineol solution with the respective powders (anode and cathode) at a mass ratio of 1:2. The inks were screen‐printed on opposing sides of the LSGM electrolyte, followed by calcination at 1050 °C for 3 h to fabricate the Ru‐SFM/Ru‐GDC || LSGM || PBSCF/GDC single cell. Ag paste was uniformly applied on the anode and cathode as the current collector. Glass ceramics was applied as the sealing material to prevent gas leakage.

The Ru‐SFM/Ru‐GDC anode was in situ reduced at 800 °C during SOFC operation in H_2_ or CH_4_ atmosphere. During fuel cell operation, humidified (3% H_2_O) with H_2_ or CH_4_ was fed to the anode at 20 mL min^−1^, while the PBSCF/GDC cathode was exposed to static air. Electrochemical measurements of the single cells were conducted from 800, 750, and 700 °C, including current–voltage and power density curves, along with electrochemical impedance spectroscopy (EIS) in a 1 MHz to 0.1 Hz frequency range. The distribution of relaxation time (DRT) method was utilized to fit the EIS data. Stability testing of the fuel cell was performed at a constant current density of 1.0 A cm^−2^ and 800 °C using the humidified CH_4_ as the fuel.

### Materials Characterization

X‐ray diffraction (XRD, Bruker D8‐Focus) was used to determine the phase structures of the as‐prepared powders. Morphology of powders and single cell were conducted by scanning electron microscopy (SEM, Hitachi SU‐8010), high‐resolution transmission electron microscopy (HRTEM, Titan G260‐300). X‐ray photoelectron spectroscopy (XPS, Kratos Axis Ultra DLD) using an Al Kα X‐ray source was applied to analyze the surface chemical states, with the C 1s peak at 284.8 eV as a calibration standard. Electron paramagnetic resonance (EPR, Bruker‐A300) was used to assess the oxygen vacancy concentration. Temperature‐programmed reduction of H_2_ (H_2_‐TPR) profiles was recorded on a Micromeritics Chemisorption Analyzer (Auto Chem II 2920) over room temperature to 900 °C. Raman spectra were collected by Alpha 300‐R Raman spectrometer in the range of 1000–2000 cm^−1^. Specific surface areas of Ru@Ru‐SFM/Ru‐GDC and SFM/GDC anodes were analyzed by Barrette–Emmett–Teller (BET) with the apparatus of ASAP 2020HD88.

### Theoretical Calculation

The Vienna Ab Initio Simulation Package (VASP)^[^ ^1^ ^]^ was used to perform density functional theory (DFT) calculations within the generalized gradient approximation (GGA‐PBE) formalism.^[^ ^2^ ^]^ The projected augmented wave (PAW) method^[^ ^3^ ^]^ described ionic cores and valence electrons using a plane wave basis set with a 450 eV kinetic energy cutoff. Partial occupancies of Kohn–Sham orbitals were permitted via Gaussian smearing (width 0.05 eV). Electronic energies were considered converged below 10^−5^ eV changes. Geometry optimizations were deemed complete when forces were below 0.05 eV Å^−1^. Grimme's DFT‐D3 methodology accounted for dispersion interactions. The gamma point sampled the Brillouin zone during structural optimizations, with bottom atomic layers fixed and the remainder relaxed. SFM (001) surface, Ru cluster@Ni‐SFM (001) and Ru cluster@Ni‐GDC (111) were built for CH_4_ conversion, respectively. The free energy of a gas phase molecule or an adsorbate on the surface was calculated by the equation *G* = *E* + ZPE − TS, where *E* is the total energy, ZPE is the zero‐point energy, *T* is the temperature in kelvin (298.15 K is set here), and *S* is the entropy. The standard hydrogen electrode (SHE) model^[^ ^4^ ^]^ was adopted in the calculations of Gibbs free energy changes (Δ*G*) of all reaction steps, which was used to evaluate the reaction barrier.

## Conflict of Interest

The authors declare no conflict of interest.

## Supporting information

Supporting Information

## Data Availability

The data that support the findings of this study are available from the corresponding author upon reasonable request.
