# Supplementary material for: Smart Dual‐Exsolved Self‐Assembled Anode Enables Efficient and Robust Methane‐Fueled Solid Oxide Fuel Cells
Source: Adv Sci (Weinh). 2023 Nov 20;11(2):2306845. doi: 10.1002/advs.202306845 (PMC10787062; doi:10.1002/advs.202306845)
Supplement: Supplementary file 1 — Supporting Information [file ADVS-11-2306845-s001.pdf]

ADVANCED SCIENCE
Open Access

Supporting Information

for *Adv. Sci.*, DOI 10.1002/advs.202306845

Smart Dual-Exsolved Self-Assembled Anode Enables Efficient and Robust Methane-Fueled Solid Oxide Fuel Cells

Feng Hu, Kongfa Chen, Yihan Ling, Yonglong Huang, Sunce Zhao, Sijiao Wang, Liangqi Gui, Beibei He and Ling Zhao**

Supporting information

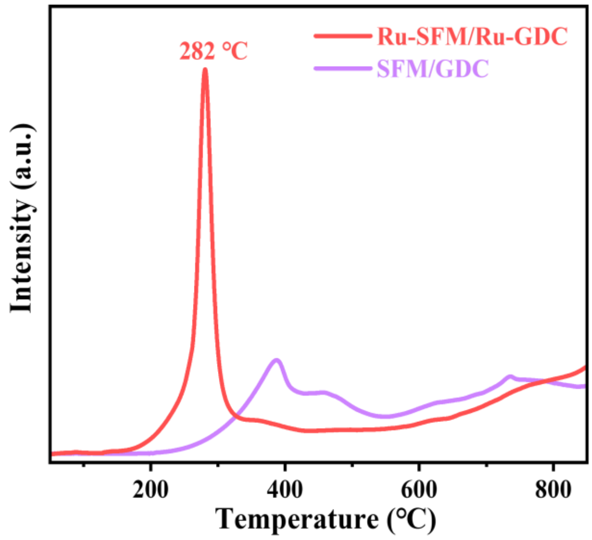

Figure S1. H_2-TPR curves of SFM/GDC and Ru-SFM/Ru-GDC powders.

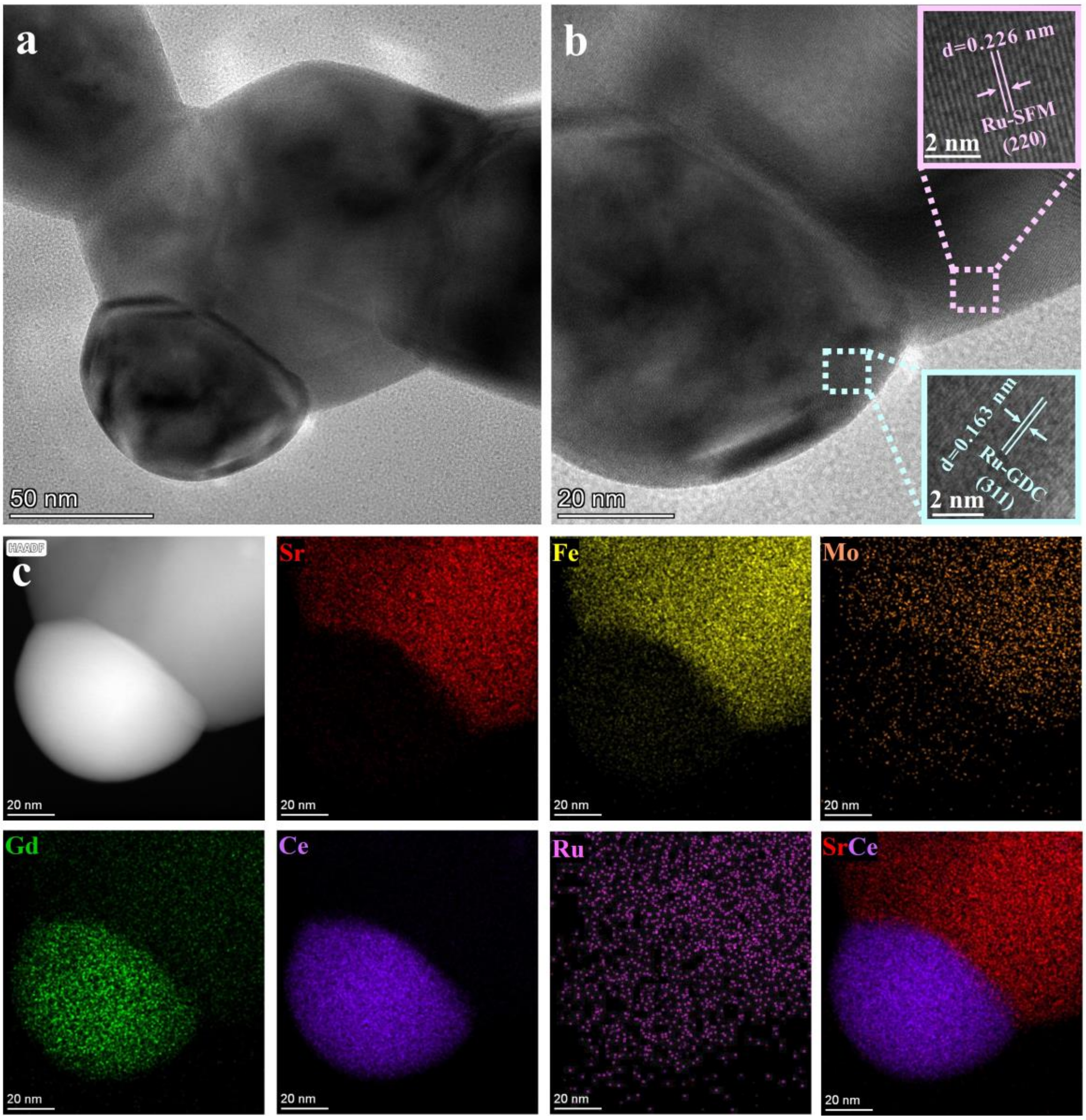

Figure S2. a) Low magnification, b) high magnification HRTEM images, c) EDS element mapping of Ru-SFM/Ru-GDC powder.

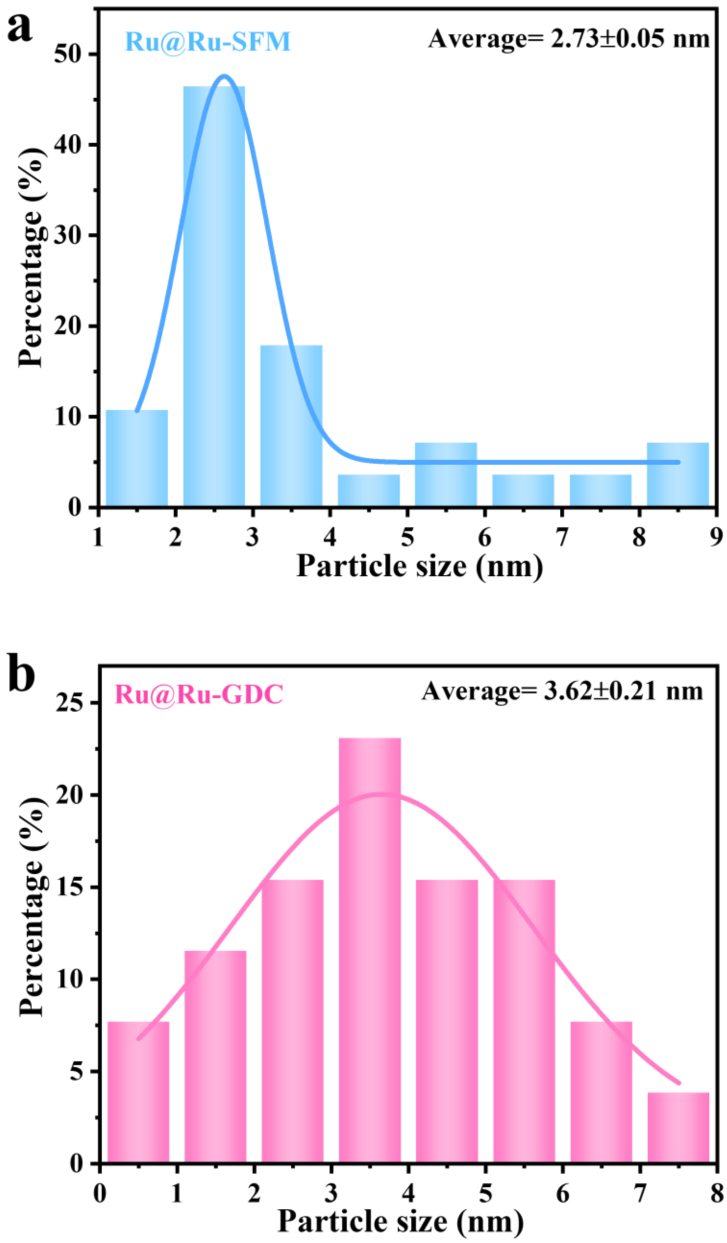

Figure S3. Particle size distribution of the exsolved Ru metal on a) Ru-SFM and b) Ru-GDC surfaces.

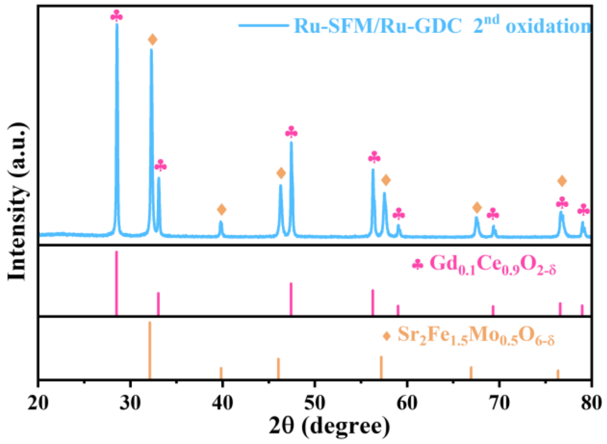

Figure S4. XRD pattern of 2nd oxidated Ru-SFM/Ru-GDC powder during redox cycle.

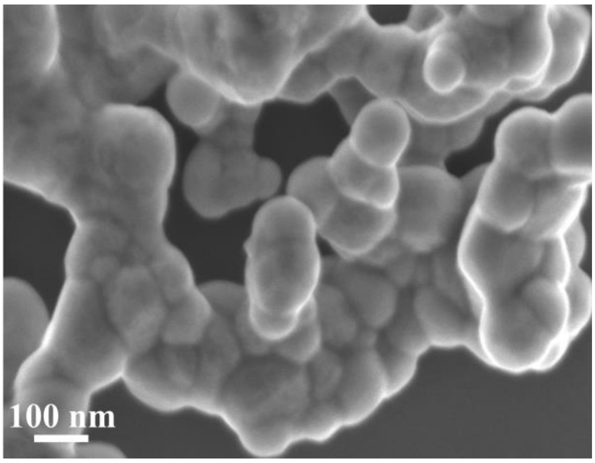

Figure S5. SEM image of 2nd oxidated Ru-SFM/Ru-GDC powder during redox cycle.

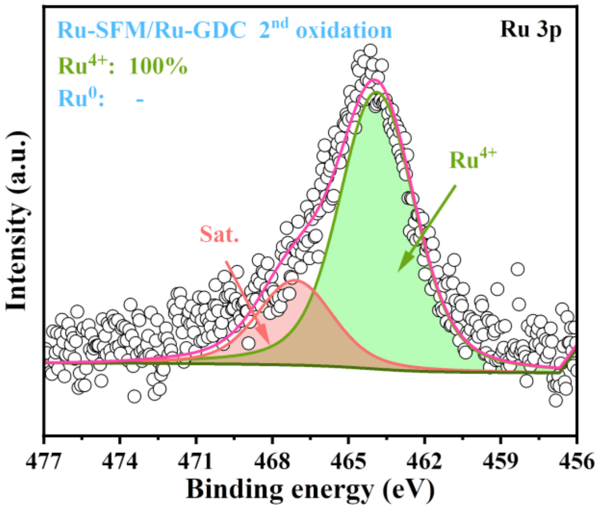

Figure S6. XPS spectrum of Ru 3p for 2nd oxidated Ru-SFM/Ru-GDC powder during redox cycle.

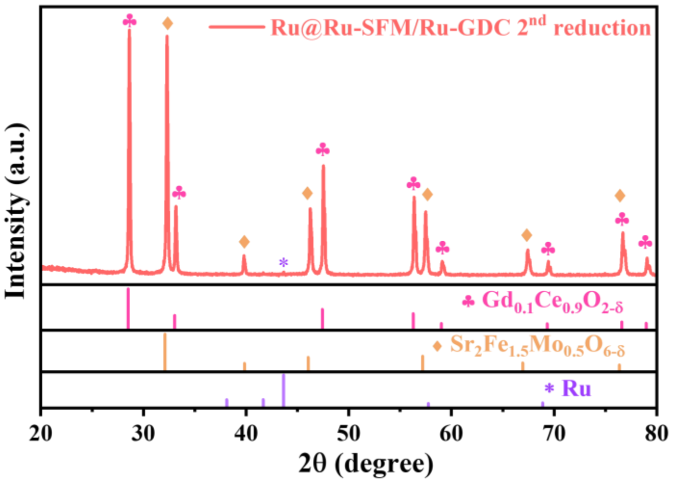

Figure S7. XRD pattern of 2nd reduced Ru@Ru-SFM/Ru-GDC powder during redox cycle.

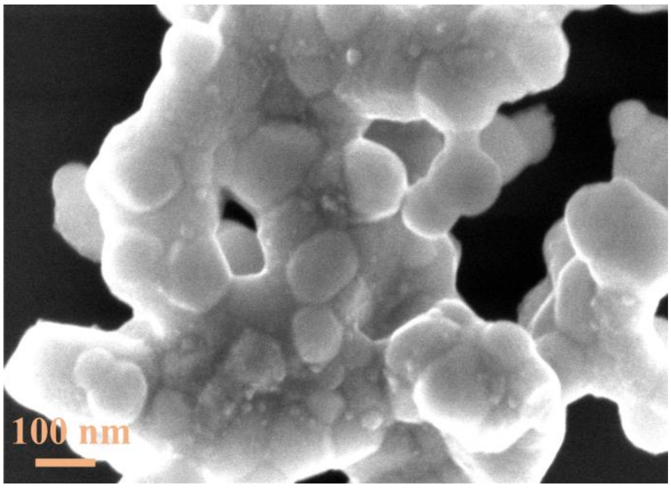

Figure S8. SEM image of 2nd reduced Ru@Ru-SFM/Ru-GDC powder during redox cycle.

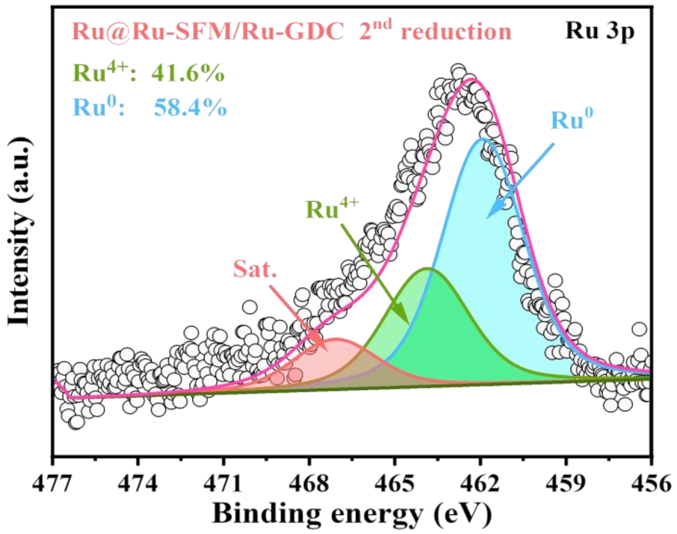

Figure S9. XPS spectrum of Ru 3p for 2nd reduced Ru@Ru-SFM/Ru-GDC powder during redox cycle.

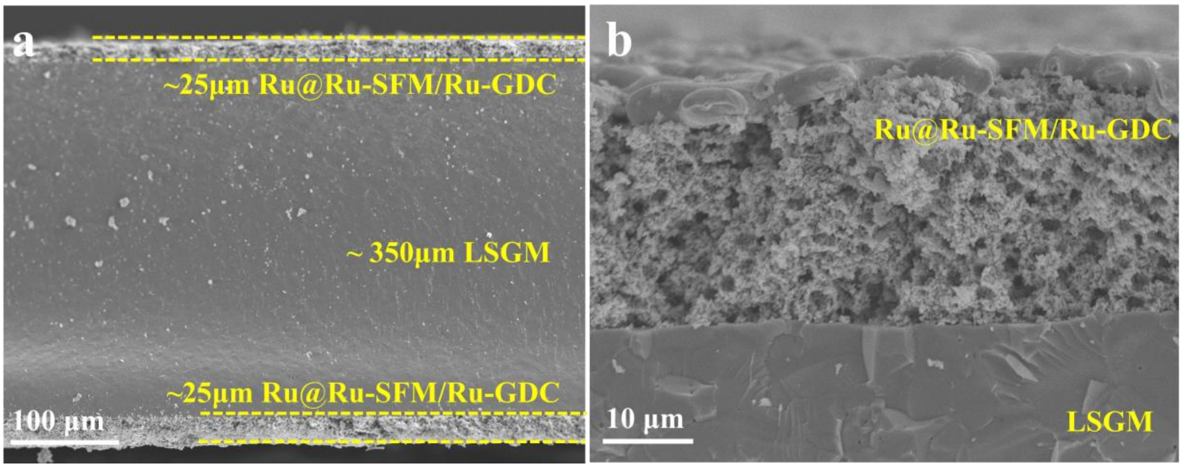

Figure S10. SEM images of a) LSGM electrolyte supported symmetrical cell, b) Ru@Ru-SFM/Ru-GDC electrode.

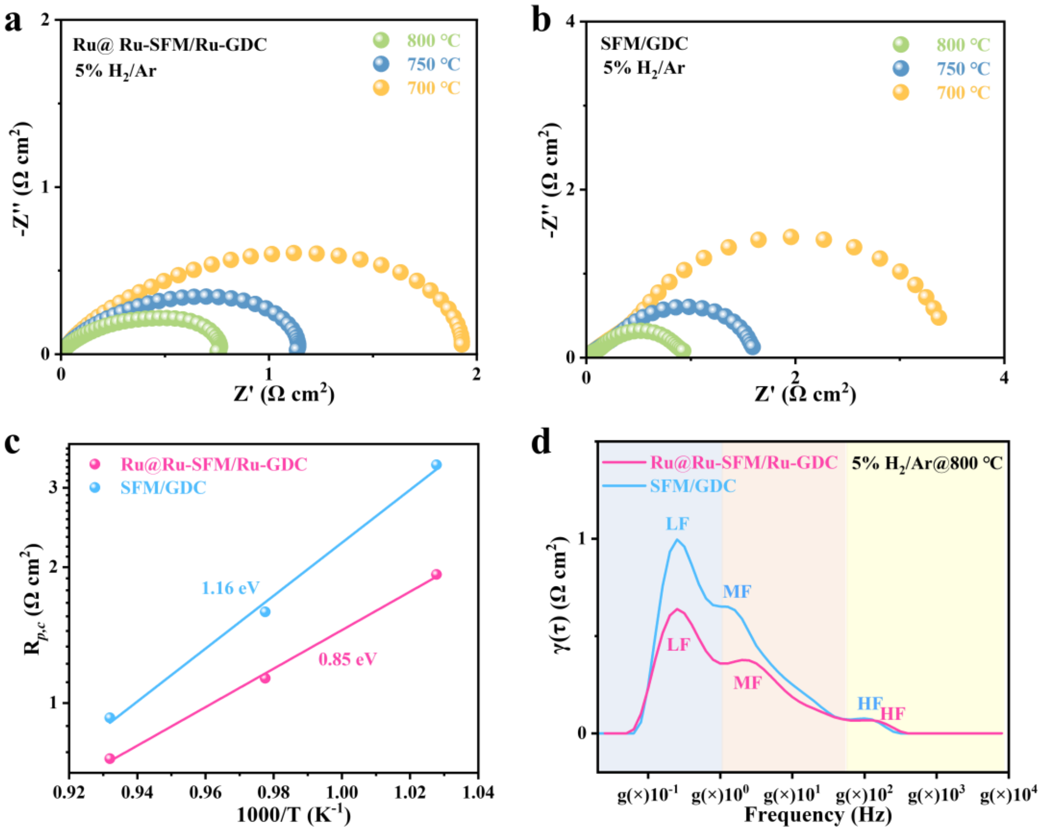

Figure S11. EIS curves of symmetrical cell testing in 5% H_2/Ar using a) Ru@Ru-SFM/Ru-GDC electrode and b) SFM/GDC electrode, c) electrode polarization resistances and d) DRT curves of SFM/GDC and Ru@Ru-SFM/Ru-GDC electrodes.

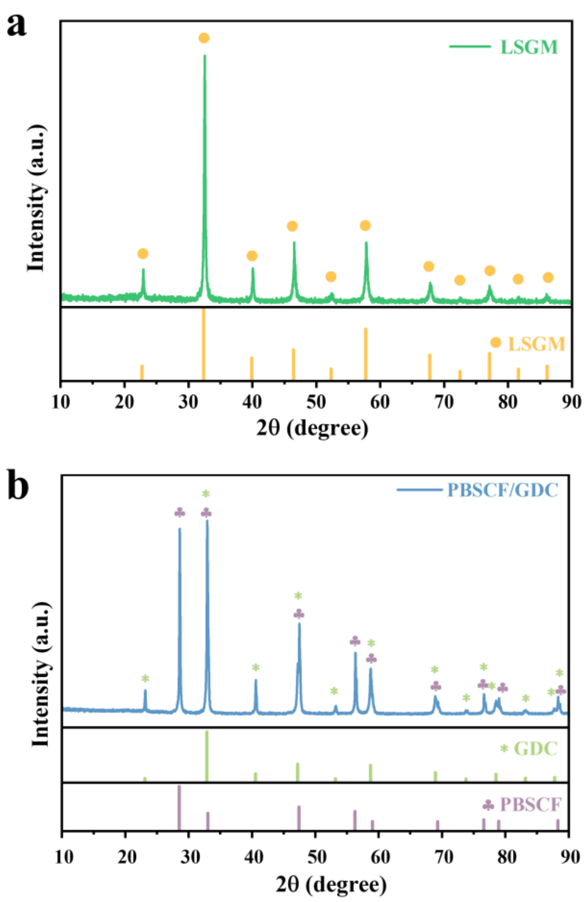

Figure S12. XRD patterns of a) LSGM electrolyte and b) PBSCF/GDC composite cathode.

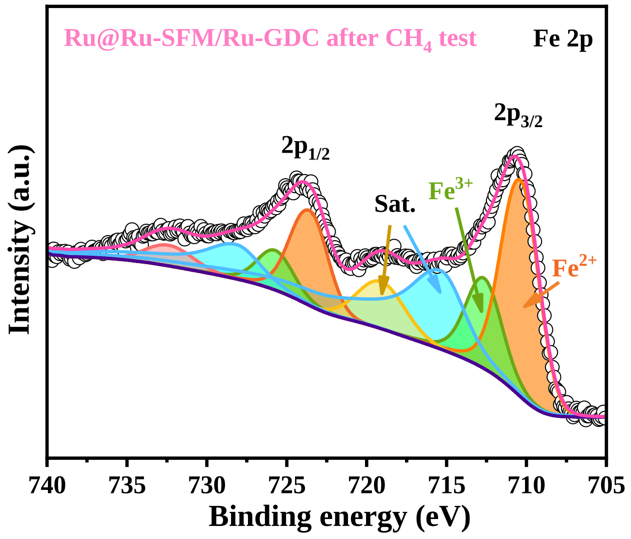

Figure S13. XPS spectrum of Fe 3p for Ru@Ru-SFM/Ru-GDC powder after long-term CH_4 test.

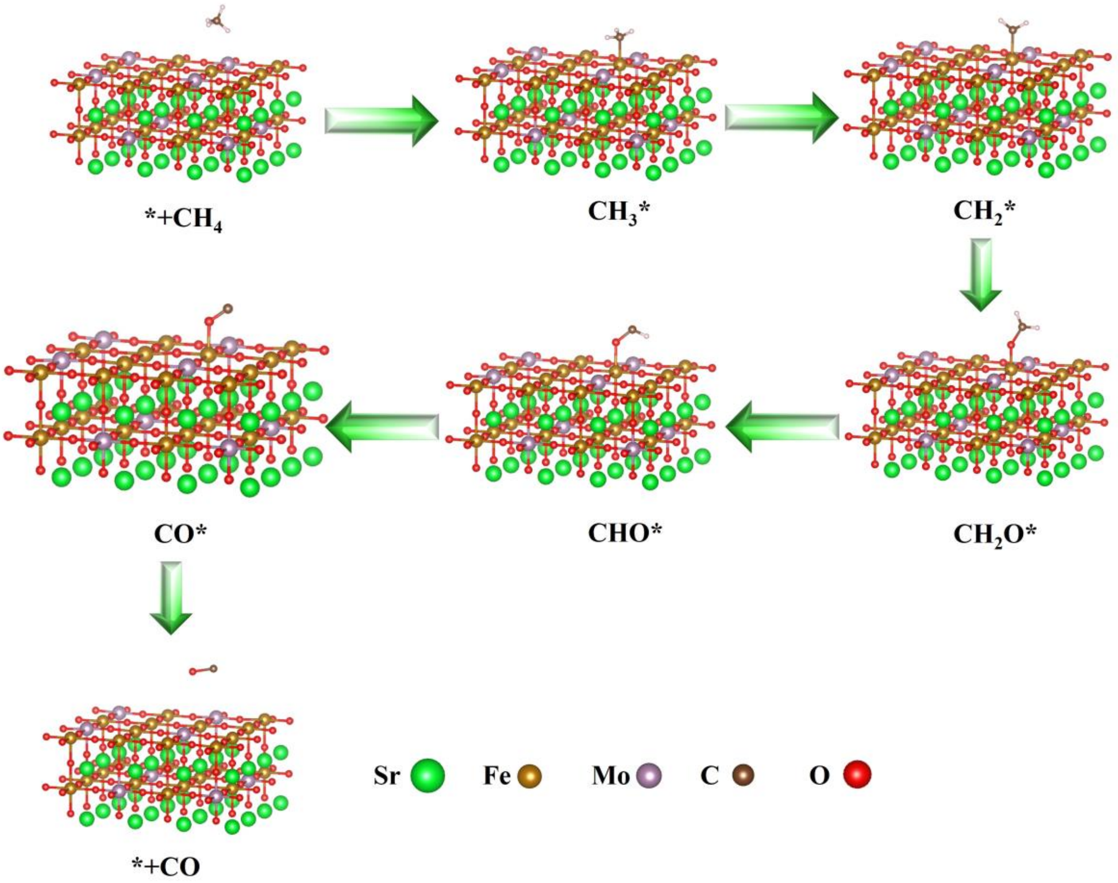

Figure S14. Adsorption configuration of reaction intermediates on SFM surface for CH_4 conversion (Sr atom: green, Fe atom: golden, Mo atom: lilac, C atom: brown, O atom: red).

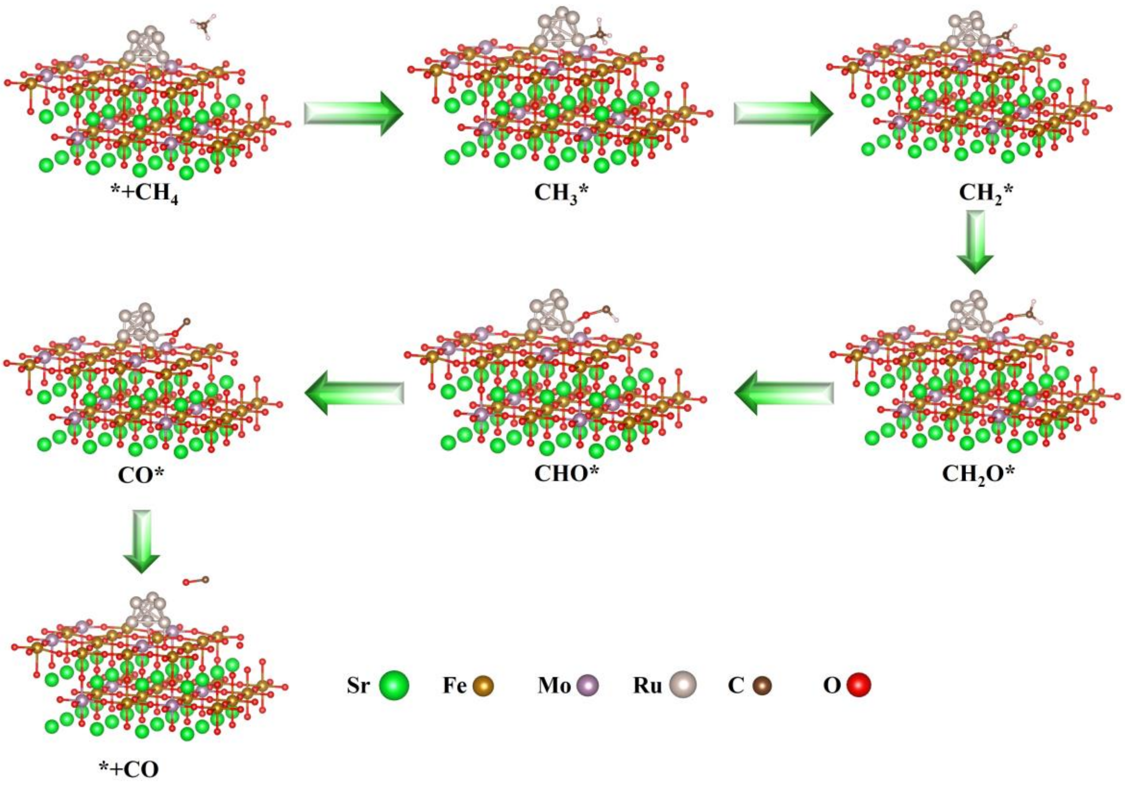

Figure S15. Adsorption configuration of reaction intermediates on Ru@Ru-SFM surface for CH_4 conversion (Sr atom: green, Fe atom: golden, Mo atom: lilac, Ru atom: grey, C atom: brown, O atom: red).

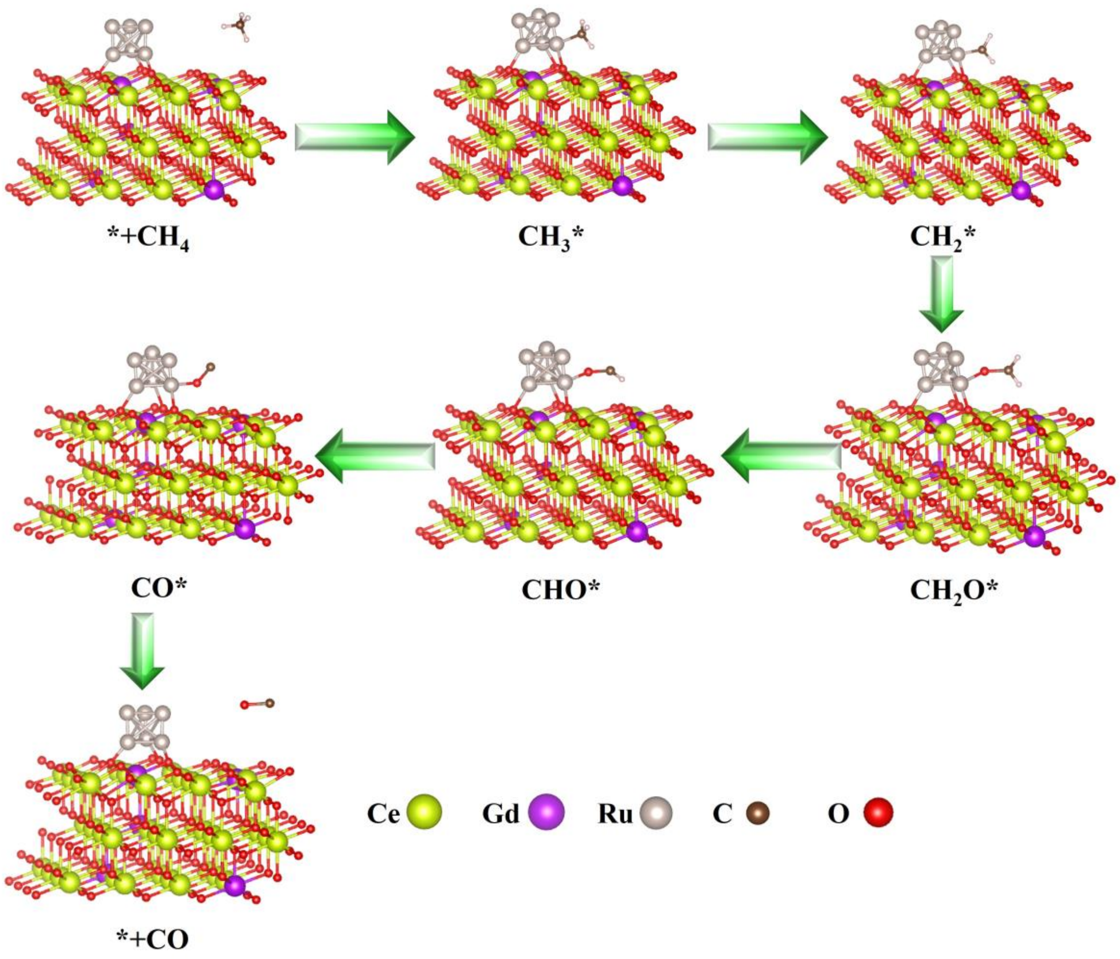

Figure S16. Adsorption configuration of reaction intermediates on Ru@Ru-GDC surface for CH_4 conversion (Ce atom: yellow, Gd atom: purple, Ru atom: grey, C atom: brown, O atom: red).

Table S1. Comoparison of element valence states in Ru@Ru-SFM/Ru-GDC anode before and after reduction derived from XPS analysis.

**Electrocatalyst**	**Ru (%)**	**Fe (%)**	**Mo (%)**	**Ce (%)**	**O (%)**
Ru-SFM/Ru-GDC	Ru^{4+}:100.0<br>Ru^{0}:-	Fe^{3+}:57.5<br>Fe^{2+}:42.5	Mo^{6+}:100.0<br>Mo^{5+}:-	Ce^{4+}:78.3<br>Ce^{3+}:21.7	CO_3^{2-}/OH^{-}:36.1<br>O_2^{2-}/O^{-}:18.7<br>$O_{lat.}$:45.2

Ru@Ru-SFM/Ru-GDC	Ru^{4+}:40.5<br>Ru^{0}:59.5	Fe^{3+}:39.0<br>Fe^{2+}:61.0	Mo^{6+}:95.0<br>Mo^{5+}:5.0	Ce^{4+}:62.0<br>Ce^{3+}:38.0	CO_3^{2-}/OH^-:39.0<br>O_2^{2-}/O^-:34.8<br>$O_{lat.}$:26.2

Table S2. Comoparison of element valence states in Ru@Ru-SFM/Ru-GDC anode during 2nd redox cycles derived from XPS analysis.

**Electrocatalyst**	**Ru (%)**	**Fe (%)**	**Mo (%)**	**Ce (%)**	**O (%)**
2nd oxidation Ru-SFM/Ru-GDC	Ru^{4+}:100.0<br>Ru^{0}:-	Fe^{3+}:57.4<br>Fe^{2+}:42.6	Mo^{6+}:100.0<br>Mo^{5+}:-	Ce^{4+}:78.6<br>Ce^{3+}:21.4	CO_3^{2-}/OH^-:36.0<br>O_2^{2-}/O^-:18.6<br>$O_{lat.}$:45.4
2nd reduction Ru@Ru-SFM/Ru-GDC	Ru^{4+}:41.6<br>Ru^{0}:58.4	Fe^{3+}:38.7<br>Fe^{2+}:61.3	Mo^{6+}:94.6<br>Mo^{5+}:5.4	Ce^{4+}:61.5<br>Ce^{3+}:38.5	CO_3^{2-}/OH^-:39.1<br>O_2^{2-}/O^-:34.8<br>$O_{lat.}$:26.1

Table S3. BET surface area of Ru@Ru-SFM/Ru-GDC and SFM/GDC anodes.

**Anode**	**S (m^2/g)**
SFM/GDC	6.38
Ru@Ru-SFM/Ru-GDC	8.80

Table S4. Summary of peak power densities of SOFCs between this work and others in H_2 at 800 °C.

**Anode**	**Electrolyte**	**Thickness (μm)**	**Cathode**	**Fuel**	**PPD ($W\ cm^2$)**	**Ref**
Ru@Ru-$Sr_2Fe_{1.5}Mo_{0.5}O_{6-\delta}$/Ru-$Gd_{0.1}Ce_{0.9}O_{2-\delta}$ (Ru@Ru-SFM/Ru-GDC)	LSGM	230	PBSCF/GDC	H_2	1.026	This work

$Sr_2Fe_{1.5}Mo_{0.5}O_{6-\delta}$ (SFM)	LSGM	320	LSCF	H_2	~0.500	[5]
$SrTi_{0.3}Fe_{0.7}O_{3-\delta}$ (STF)	LSGM	500	LSCF	H_2	0.700	[6]
$Sr_2Fe_{1.5}Mo_{0.5}O_{6-\delta}$-R (SFM-R)	LSGM	230	LSCF/SDC	H_2	1.240	[7]
Ru@$Ce_{0.2}Sr_{0.8}Fe_{0.95}Ru_{0.05}O_3$ (Ru@Ce20SFR)	LSGM	320	Ce20SFR	H_2	0.850	[8]
CoFe-Pd@$La_{0.6}Sr_{0.4}Co_{0.15}Fe_{0.8}Pd_{0.05}O_{3-\delta}$ (CoFe-Pd@LSCFP)	LSGM	300	LSCF/GDC	H_2	1.680	[9]
Fe-Co-Ni@$Sr_2FeCo_{0.2}Ni_{0.2}Mo_{0.6}O_{6-\delta}$ (Fe-Co-Ni@SFCNM)	LSGM	270	LSCF/GDC	H_2	0.850	[10]
RuFe@Sr($Ti_{0.3}Fe_{0.7}Ru_{0.07}$)$O_{3-\delta}$ (RuFe@STF-Ru)	ScSZ	140	STF	H_2	0.400	[11]
Ni@$Sr_3Fe_{1.3}Mo_{0.5}Ni_{0.2}O_{7-\delta}$-$Sm_{0.1}Ce_{0.9}O_{2-\delta}$ (Ni@RP-SFMN-SDC)	LSGM	400	LSCF/GDC	H_2	0.409	[12]
$FeNi_3$@$Sr_2FeMo_{0.65}Ni_{0.35}O_{6-\delta}$ ($FeNi_3$@SFMN)	LSGM	300	LSCF	H_2	0.790	[13]
NiFe@$La_{0.6}Ce_{0.1}Sr_{0.3}Fe_{0.9}Ni_{0.1}O_{3-\delta}$ (NiFe@CLSFN)	LSGM	300	CLSFN	H_2	0.675	[14]

LSGM, (La, Sr)$Ga_{0.8}Mg_{0.2}O_{3-\delta}$; PBSCF, $PrBa_{0.5}Sr_{0.5}Co_{1.5}Fe_{0.5}O_{6-\delta}$; GDC, (Gd, Ce) $O_{2-\delta}$; LSCF, (La, Sr) (Co, Fe) $O_{3-\delta}$; SDC, $Sm_{0.2}Ce_{0.8}O_{1.9}$; ScSZ, Sc_2O_3-ZrO_2.

Table S5. Summary of peak power densities and stabilities of SOFCs between this work and others in CH_4 at 800 °C.

**Anode**	**Electrolyte**	**Thickness (μm)**	**Cathode**	**Fuel**	**PPD ($W\ cm^{-2}$)**	**Stability (h)**	**Ref**
Ru@Ru-$Sr_2Fe_{1.5}Mo_{0.5}O_{6-\delta}$/Ru-$Gd_{0.1}Ce_{0.9}O_{2-\delta}$	LSGM	230	PBSCF/GDC	CH_4	0.63	200	This work

(Ru@Ru-SFM/Ru-GDC)							
$Sr_2Fe_{1.5}Mo_{0.5}O_{6-\delta}$ (SFM)	LSGM	320	LSCF	CH_4	0.05	-	[5]
Sr_2ZnMoO_4 (R-SZMO)	LSGM	300	LSCF	CH_4	0.33	110	[15]
Co@$Sr_2Fe_{1.3}Co_{0.2}Mo_{0.5}O_{6-\delta}$ (Co@SFCM)	LSGM	170	LSCF	CH_4	0.15	200	[16]
Co-Ni-Mo/$Sr_2FeMoO_{6-\delta}$ (CNM/SFM)	LSGM	300	CNM/SFM	CH_4	~0.48	24	[17]
CoFe@$La_{0.5}Ba_{0.5}Mn_{0.8}Fe_{0.1}Co_{0.1}O_{3-\delta}$ (CoFe@LBMFC-2)	LSGM	300	BSCF	CH_4	0.26	200	[18]
Pr_6O_{11}-$PrBaMn_2O_{5+\delta}$ (Pr-PBMO)	YSZ	-	Pr-PBMO	CH_4	0.23	130	[19]
Ni@$La_{0.4}Sr_{0.4}Ti_{0.85}Ru_{0.07}Ni_{0.08}O_{3-\delta}$ (Ni@L0.4STRN)	LSGM	500	LSCF	CH_4	0.45	25	[20]
Ni@$(La_{0.2}Sr_{0.8})_{0.925}Ti_{0.55}Mn_{0.35}Ni_{0.1}O_{3-\delta}$ (Ni@LSTMN)	LSGM	350	LSCF	CH_4	0.39	100	[21]
NiFe@$La_{0.6}Ce_{0.1}Sr_{0.3}Fe_{0.9}Ni_{0.1}O_{3-\delta}$ (NiFe@CLSFNi)	LSGM	300	CLSFNi	CH_4	0.52	14	[14]
FeCoNiCuAl-$Sm_{0.2}Ce_{0.8}O_2$ (FeCoNiCuAl-SDC)	LSGM	300	LSCF	CH_4	0.37	20	[22]

LSGM, (La, Sr) $Ga_{0.8}Mg_{0.2}O_{2-\delta}$; PBSCF, $PrBa_{0.5}Sr_{0.5}Co_{1.5}Fe_{0.5}O_{6-\delta}$; GDC, (Gd, Ce) $O_{2-\delta}$; LSCF, (La, Sr) (Co, Fe) $O_{3-\delta}$; BSCF, $Ba_{0.5}Sr_{0.5}Co_{0.5}Fe_{0.5}O_{6-\delta}$; YSZ, Y_2O_3-ZrO_2.

References

[1] a) G. Kresse and J. Furthmuller, *Computational Materials Science* **1996**, *6*, 15-50; b) G. Kresse and J. Furthmuller, *Physical Review B* **1996**, *54*, 11169-11186.
[2] J. P. Perdew, K. Burke and M. Ernzerhof, *Physical Review Letters* **1996**, *77*, 3865-3868.
[3] a) G. Kresse and D. Joubert, *Physical Review B* **1999**, *59*, 1758-1775; b) P. E. Blochl, *Physical Review B* **1994**, *50*, 17953-17979.

[4] J. K. Norskov, J. Rossmeisl, A. Logadottir, L. Lindqvist, J. R. Kitchin, T. Bligaard and H. Jonsson, *Journal of Physical Chemistry B* **2004**, *108*, 17886-17892.

[5] Q. A. Liu, X. H. Dong, G. L. Xiao, F. Zhao and F. L. Chen, *Advanced Materials* **2010**, *22*, 5478-5482.

[6] T. Zhu, D. E. Fowler, K. R. Poeppelmeier, M. Han and S. A. Barnett, *Journal of the Electrochemical Society* **2016**, *163*, F952-F961.

[7] X. A. Xi, J. W. Liu, W. Z. Luo, Y. Fan, J. J. Zhang, J. L. Luo and X. Z. Fu, *Advanced Energy Materials* **2021**, *11*, 1614-6832.

[8] B. Li, S. He, J. Li, X. Yue, J. T. S. Irvine, D. Xie, J. Ni and C. Ni, *ACS Catalysis* **2020**, *10*, 14398-14409.

[9] K. J. Kim, C. Lim, K. T. Bae, J. J. Lee, M. Y. Oh, H. J. Kim, H. Kim, G. Kim, T. H. Shin, J. W. Han and K. T. Lee, *Applied Catalysis B-Environmental* **2022**, *314*, 121517.

[10] C. Li, Y. Deng, L. Yang, B. Liu, D. Yan, L. Fan, J. Li and L. Jia, *Advanced Powder Materials* **2023**, *2*, 100133.

[11] A. Donazzi, T. A. Schmauss and S. A. Barnett, *Journal of Power Sources* **2022**, *551*, 232215.

[12] X. Zhang, Y. Tong, T. Liu, D. Zhang, N. Yu, J. Zhou, Y. Li, X. K. Gu and Y. Wang, *SusMat* **2022**, *2*, 487-501.

[13] F. Liu, L. L. Zhang, G. Z. Huang, B. B. Niu, X. R. Li, L. Wang, J. Zhao and Y. Jin, *Electrochimica Acta* **2017**, *255*, 118-126.

[14] L. Bian, C. Duan, L. Wang, R. O'Hayre, J. Cheng and K.-C. Chou, *Journal of Materials Chemistry A* **2017**, *5*, 15253-15259.

[15] Y. H. Su, T. Wei, Y. N. Li, B. Y. Yin, Y. Huan, D. H. Dong, X. Hu and B. L. Huang, *Journal of Materials Chemistry A* **2021**, *9*, 5067-5074.

[16] Y. R. Yang, Y. R. Wang, Z. B. Yang, Z. Lei, C. Jin, Y. D. Liu, Y. H. Wang and S. P. Peng, *Journal of Power Sources* **2019**, *438*, 0378-7753.

[17] M. K. Rath and K. T. Lee, *Electrochimica Acta* **2016**, *212*, 678-685.

[18] N. J. Hou, T. T. Yao, P. Li, X. L. Yao, T. Gan, L. J. Fan, J. Wang, X. J. Zhi, Y. C. Zhao and Y. D. Li, *ACS Applied Materials & Interfaces* **2019**, *11*, 6995-7005.

[19] Y. H. Gu, Y. L. Zhang, Y. F. Zheng, H. Chen, L. Ge and L. C. Guo, *Applied Catalysis B-Environmental* **2019**, *257*, 117868.

[20] Y. W. Tang, H. C. Wang, R. C. Wang, Q. S. Liu, Z. X. Yan, L. L. Xu and X. J. Liu, *ACS Applied Materials & Interfaces* **2022**, *60*, 7826-7834.

[21] X. X. Yang, W. Sun, M. J. Ma, C. M. Xu, R. Z. Ren, J. S. Qiao, Z. H. Wang, S. Y. Zhen and K. N. Sun, *Industrial & Engineering Chemistry Research* **2021**, *60*, 7826-7834.

[22] D. Z. Chen, Y. Huan, G. J. Ma, M. Y. Ma, X. J. Wang, X. Y. Xie, J. F. Leng, X. Hu and T. Wei, *ACS Applied Energy Materials* **2023**, *6*, 1076-1084.
